# Draft genome sequence of *Clostridium tetanomorphum* DSM 4474^T^ isolated from a septic wound

**DOI:** 10.1128/mra.00842-25

**Published:** 2025-11-04

**Authors:** Ally Watkins, Charles Walker Cano, Emma Hoover, Nicholas Peterson, Stefan Spring, Markus Goker, Natalia Ivanova, Rekha Seshadri, Tricia A. Van Laar

**Affiliations:** 1 Department of Biological Sciences, California State University, Stanislaus 14674, Turlock, California, USA; 2 Leibniz Institute DSMZ-German Collection of Microorganisms and Cell Cultures GmbH, Braunschweig, Germany; 3 Lawrence Berkeley National Laboratory, DOE Joint Genome Institute 1666, Berkeley, California, USA; University of Pittsburgh School of Medicine, Pittsburgh, Pennsylvania, USA

**Keywords:** opportunistic infections, *Clostridium*, butanol

## Abstract

We report the genome sequence of *Clostridium tetanomorphum* DSM 4474^T^ isolated from a septic wound. In addition to its potential as a human pathogen, this species may be useful in biofuel production due to its ability to synthesize butanol without the byproduct acetone. The genome is 4,374,054 bp.

## ANNOUNCEMENT

*Clostridium tetanomorphum*, a Gram-positive, spore-forming anaerobe, produces butanol with minimal acetone, a significant byproduct of other *Clostridium* species (1, 2). Genetic characterization of butanol production can inform methods to synthesize butanol (1), a potential low-polluting alternative to crude oil (3). This strain was first isolated in 1916 by M. Robertson (4, 5) and repurified in 1922 by the deep agar colony method (6).

The genome of *C. tetanomorphum* DSM 4474^T^ was sequenced in Phase IV of the Genomic Encyclopedia of Type Strains to characterize organisms which can provide a foundation for future genomic and metagenomic studies. Liquid DSM medium 104c under 80% N_2_ and 20% CO_2_ gas atmosphere at 37°C was inoculated with a lyophilized sample and incubated for 24 h. Genomic DNA was extracted from an estimated 40–80 mg pellet using the MasterPure Gram-positive DNA Purification Kit (Epicentre). DNA was sheared to 300 bp using a Covaris LE220 focused-ultrasonicator, and fragments were end-repaired, A-tailed, and ligated with Illumina compatible sequencing adaptors from IDT. Plate-based DNA library preparation for Illumina sequencing was performed on the PerkinElmer Sciclone NGS robotic liquid handling system using Kapa Biosystems library preparation kit (Roche). The genome was sequenced using Illumina NovaSeq S4 technology, following a 2 × 150 indexed run protocol, generating 6,298,278 reads. Default parameters for all software were used except where otherwise noted. Quality control and trimming were performed using BBTools (7). The genome was assembled using SPAdes v3.14.1 (--phred-offset 22 --cov-cutoff auto -t 16 m 64 --careful -k 25,55,95) (8). The NCBI Prokaryotic Genome Annotation Pipeline (PGAP) was used to perform the standard structural and functional annotation before addition to the Integrated Microbial Genome (IMG) system for further analysis (9, 10). CheckM2 determined the genome to be 100% complete with 1.42% contamination (11).

**Fig 1:**
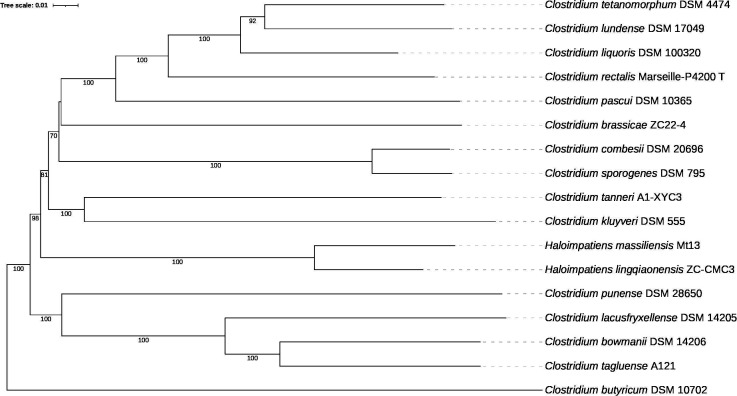
Tree inferred with FastME 2.1.6.1 (19) from whole-proteome-based GBDP distances. The branch lengths are scaled via GBDP distance formula *d_5_*. Branch values are GBDP pseudo-bootstrap support values > 60% from 100 replications, with an average branch support of 92.8%. The tree was midpoint-rooted (20) and visualized using the Interactive Tree of Life (iTOL) v. 6 (21).

The draft genome of *C. tetanomorphum* DSM 4474^T^ is 4,374,054 bp with an average fold coverage of ~215× and a G + C content of 28.84% (Table 1). The Type Strain Genome Server (TYGS) (12) (Fig. 1) identified *Clostridium liquoris* DSM 100320^T^ and *C. lundense* DSM 17049^T^ as the closest relatives to *C. tetanomorphum* supported by the Genome Taxonomy Database (GTDB) (13) which lists these species as members of their own genus (*Clostridium_AO*). We calculated the average nucleotide identity (ANI) with fastANI v1.34 (14) and found similarity to *C. lundense* at 83.7% and to *C. liquoris* at 81.3%. We confirmed the presence of butanol production genes in this strain using BLASTn. We used PathogenFinder2 (v. 0.4.1) (15) and found a mean prediction of 76.88% of pathogenic capacity. Unique virulence factors involved in cell motility, toxin production, and adhesion were detected by querying the Virulence Factor Database (16) with BLAST. Comprehensive Antibiotic Resistance Database (CARD, v. 4.0.0) (17) was queried using Resistance Gene Identifier (RGI). This identified genes conferring resistance to glycopeptides and fluoroquinolones. antiSMASH (v. 8) (18) predicted genes involved in antimicrobial peptide biosynthesis and quorum sensing. This strain’s dual potential as an opportunistic pathogen and industrially relevant organism supports future study into its metabolic and pathogenic capabilities.

**TABLE 1:** Genomic features of *C. tetanomorphum* DSM 4474

Feature	Finding
Genome/Scaffold length (bp)	4,374,054
No. of scaffolds	56
GC content (%)	28.84
No. of genes	4,433
No. of rRNAs	10
No. of tRNAs	69
Scaffold N50 (bp)	152,605
Average fold coverage (x)	214.955
Number of protein-coding genes	4,250

## Data Availability

This Whole Genome Shotgun project has been deposited in DDBJ/ENA/GenBank under the accession number JAGGKA000000000. The version described in this paper is the first version, JAGGKA010000000. The raw reads have been deposited in the NCBI Sequence Read Archive (SRA) under the accession number SRX20649568. Additional data can be explored or downloaded from the JGI Integrated Microbial Genomes with Microbiomes (IMG/M) portal using the taxon ID 2913338184.
